# The complete mitogenome of scaly thrush *Zoothera aurea* (Passeriformes, Turdidae)

**DOI:** 10.1080/23802359.2020.1787267

**Published:** 2020-07-24

**Authors:** Jiaqi Li, Keer Miao, Xi Wu, Qi Wang, Chaochao Hu, Peng Li

**Affiliations:** a Nanjing Institute of Environmental Sciences, Ministry of Ecology and Environment, Nanjing, China; b Jiangsu Key Laboratory for Biodiversity and Biotechnology, College of Life Sciences, Nanjing Normal University, Nanjing, China; c Nanjing Lukou International Airport, Nanjing, China

**Keywords:** Mitogenome, Passeriformes, Turdidae, *Zoothera aurea*

## Abstract

The complete mitochondrial genome of scaly thrush *Zoothera aurea* was obtained by next-generation sequencing. The circular genome was 16,712 bp in length, consisting of 13 protein-coding genes, 22 transfer RNA genes, two ribosomal RNA genes, and a control region. The overall nucleotide composition was A: 29.67%, T: 23.32%, C: 32.21%, and G: 14.80%. Nine genes were encoded on the light strand, and the remaining 28 genes were encoded on the heavy strand. Most of the PCGs began with the ATG as the start codon, and six kinds of termination codons were used in this mitogenome. This study improves our understanding of the mitogenomic characteristics and its phylogenetic relationships within Turdidae.

The scaly thrush *Zoothera aurea* (Passeriformes, Turdidae) has an extremely large range, and breeds mainly in East Asia and Siberia in moist coniferous forests. Non-breeders are thought to move to the Himalayan foothills, lowlands of the north east Indian subcontinent, and south-east Asia. Despite the fact that the population trend appears to be decreasing, it is evaluated as Least Concern (BirdLife International 2020). With the difference in morphology, ecology, behavior, taxonomic treatment of the *Zoothera dauma* species complex is highly variable and many species were recognized (Weir 2018). However, the basic genetics data of *Z. aurea* is still uncertain. In this study, we sequenced the complete mitogenome of *Z. aurea* to further understanding the mitogenomic characteristics and its phylogenetic relationships within Turdidae.

The muscle sample of *Z. aurea* was collected from the Lukou International Airport, Nanjing, Jiangsu Province, China (31°43′47″ N, 118°52′26″ E). The voucher specimen was stored in absolute ethanol, which was preserved at −20 °C in our laboratory at Nanjing Normal University, Jiangsu, China (specimen voucher: NJNU-Zaur04). The overall DNA was extracted by standard phenol-chloroform methods (Sambrook et al. 1989). On the Illumina HiSeq 2000 platform, the sequencing libraries with average insertion sizes of about 300 bp has been prepared, and then sequenced into a coupled sequence at 150 bp (original data is about 12 Gb) (Illumina, San Diego, CA, USA). Genius software 9.1.4 was used for analyzing sequence quality, data pruning, and re-editing (Kearse et al. 2012). The positions of protein-coding genes, ribosomal RNA (rRNAs) and transfer RNA (tRNAs) are predicted by the MITOS Web server and spotted by mitochondrial gene alliances with other mitogenome of Turdidae species (Bernt et al. 2013).

The length of circular mitochondrial genome is 16,712 bp, with 13 protein-coding genes (PCGs), two ribosomal RNAs (12S rRNA and 16S rRNA), 22 transfer RNA genes, and a non-coding region. The note on the mitogenome of *Z. aurea* has been imported into GenBank (MT527192). The total nucleotide composition was A: 29.67%, T: 23.32%, C: 32.21%, and G: 14.80%. Among the 37 genes, nine genes (tRNA^Gln^, tRNA^Ala^, tRNA^Asn^, tRNA^Cys^, tRNA^Tyr^, tRNA^Ser^, ND6, tRNA^Pro^ and tRNA^Glu^) were encoded on the light strand, and the remaining 28 genes were encoded on the heavy strand. The complete length of 13 protein-coding genes is 11,404 bp accounting for 65.46% of the total genome. Most of the PCGs began with the ATG as the start codon.

We used the maximum-likelihood (ML) algorithm in MEGA X to analyze the phylogenetic in Turdidae with other nine species based on mitochondrial complete genome (Kumar et al. 2018). The mitogenomes sequences were aligned using Muscle in MEGA X, and subsequently edited and trimmed (Kumar et al. 2018). The phylogenetic tree was constructed using *Ficedula zanthopygia*, *Passer montanus* as outgroups. The total length of 15,417 bp were used for phylogenetic analyses, and ML phylogeny was inferred under the HKY + *G* model for 1000 bootstraps (Tamura et al. 2013). The phylogenetic analysis (Figure 1) revealed well-supported mitochondrial branches. This study raises our awareness of the evolution of mitochondrial DNA in Turdidae.

**Figure 1.**
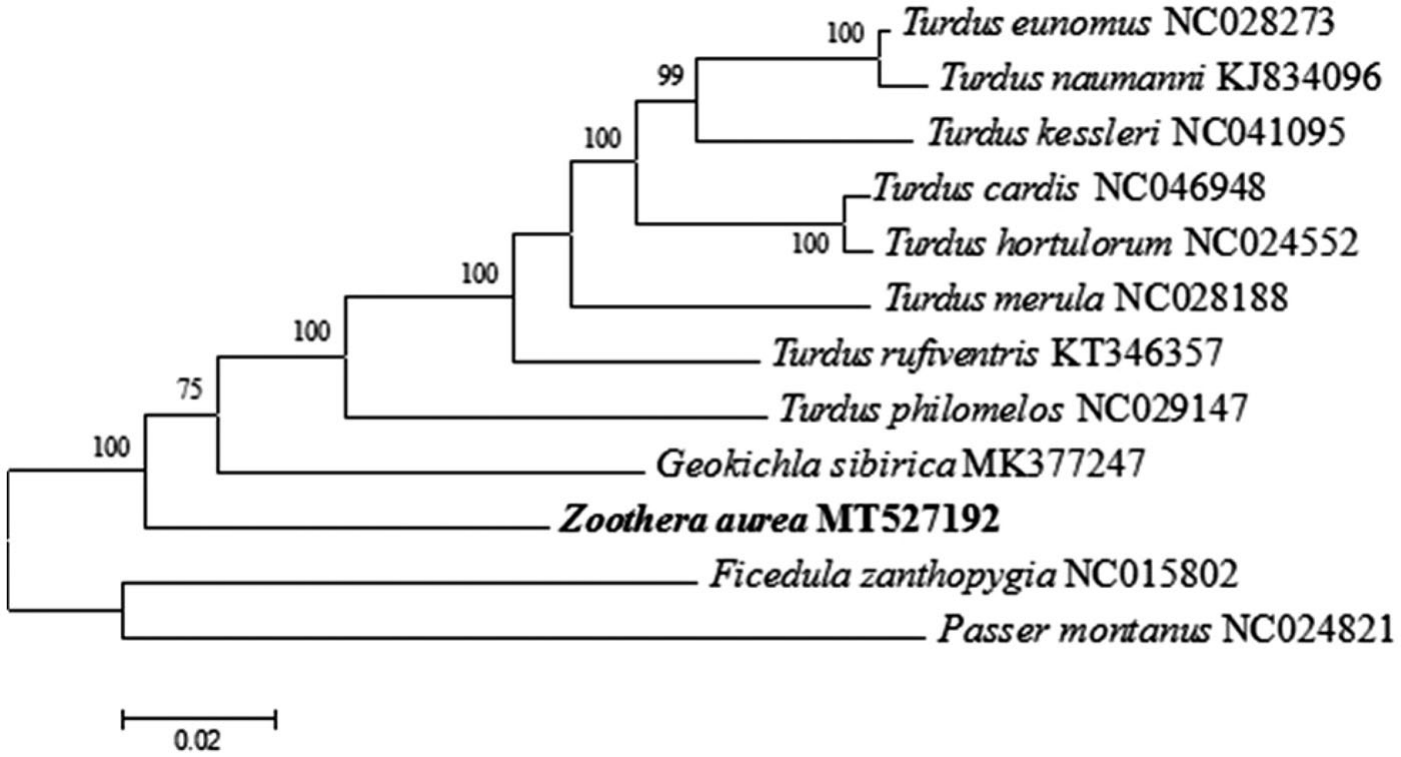
Phylogeny of *Zoothera aurea* and closely related nine mitochondrial sequences constructed using the maximum-likelihood method based on complete mitogenome. Numbers above each branch is the ML bootstrap support. GenBank accession numbers of each species are shown.

## Data Availability

The data that support the findings of this study are openly available in GenBank of NCBI at https://www.ncbi.nlm.nih.gov, reference number MT527192.
